# Multiple Reaction Monitoring–Mass Spectrometric Immunoassay Analysis of Parathyroid Hormone Fragments with Vitamin D Deficiency in Patients with Diabetes Mellitus

**DOI:** 10.3390/proteomes12040030

**Published:** 2024-10-14

**Authors:** Hicham Benabdelkamel, Refat M. Nimer, Afshan Masood, Maha Al Mogren, Anas M. Abdel Rahman, Assim A. Alfadda

**Affiliations:** 1 Proteomics Resource Unit, Obesity Research Center, College of Medicine, King Saud University, Riyadh 11451, Saudi Arabia; hbenabdelkamel@ksu.edu.sa (H.B.); afsmasood@ksu.edu.sa (A.M.); 2 Department of Medical Laboratory Sciences, Jordan University of Science and Technology, Irbid 22110, Jordan; rmnimer@just.edu.jo; 3 Metabolomics Section, Department of Clinical Genomics, Center for Genome Medicine, King Faisal Specialist Hospital and Research Centre (KFSHRC), Riyadh 11211, Saudi Arabia; mahamogren@gmail.com; 4 Department of Biochemistry and Molecular Medicine, College of Medicine, Alfaisal University, Riyadh 11533, Saudi Arabia; 5 Department of Medicine, College of Medicine, King Saud University, Riyadh 11451, Saudi Arabia

**Keywords:** diabetes, vitamin D deficiency, mass spectrometric immunoassay (MSIA), multiple reaction monitoring, parathyroid hormone

## Abstract

Current immunoassay techniques for analyzing clinically relevant parathyroid hormone (PTH) circulating fragments cannot distinguish microheterogeneity among structurally similar molecular species. This hinders the identification of molecular species and the capture of target analyte information. Since structural modifications are important in disease pathways, mass spectrometry can detect, identify, and quantify heterogeneous ligands captured by antibodies. We aimed to create a sensitive and selective multiple reaction monitoring–mass spectrometric immunoassay analysis (MRM-MSIA)-based method for detecting and quantifying PTH fragments or proteoforms for clinical research. Our study established MRM transitions using triple-quadrupole tandem mass spectrometry for the signature peptides of five PTH fragments. This method was validated according to FDA guidelines, employing the mass spectrometric immunoassay (MSIA) protocol to bolster detection selectivity and sensitivity. This validated approach was applied by analyzing samples from type 2 diabetes mellitus (T2DM) patients with and without vitamin D deficiency. We found serum PTH fragments associated with vitamin D deficiency in patients with and without T2DM. We developed and validated the MRM-MSIA technique specifically designed for the detection and quantification (amino acid (aa38–44), (aa45–51), and (aa65–75)) of these fragments associated with vitamin D deficiency and T2DM. This study is the first to accurately quantify plasma PTH fragments using MRM-MSIA, demonstrating its potential for clinical diagnostics.

## 1. Introduction

The parathyroid hormone (PTH) is a peptide hormone that, along with 25-hydroxyvitamin D (VitD), is important for regulating bone metabolism and calcium homeostasis. Aside from their action on the bone, previous studies have linked altered levels of PTH and Vit D with increased insulin resistance and the risk of developing metabolic disease [1,2,3]. Metabolic diseases, including type 2 diabetes mellitus (T2DM), are known to cause metabolic derangements that potentially affect bone mineral metabolism and the parathyroid axis. Several studies have extensively documented the link between a deficiency in vitamin D and the development of prediabetes and diabetes [4,5,6]. Vitamin D deficiency can be categorized as deficient if levels are less than 50 nmol/L, insufficient between 50 and 70 nmol/L, and sufficient if more than 75 nmol/L [7]. The concentration of extracellular calcium primarily regulates the release of PTH. However, it may also be influenced by blood phosphate and 1,25(OH)2D levels. When vitamin D levels are low, it can lead to decreased calcium absorption in the intestines. Low vitamin D can also result in reduced calcium levels in the blood. In response to low blood calcium, the parathyroid glands release PTH [8], resulting in an inverse relationship between the Vit D and PTH levels, clinically referred to as secondary hyperparathyroidism [9]. Although this is seen in many cases, not all Vit D deficient individuals manifest with increased PTH levels, and about 10% of diabetic patients with severe vitamin D deficiency have a low PTH [10]. This raises the question of the measurement method and the proteoforms detected.

**Figure 1:**
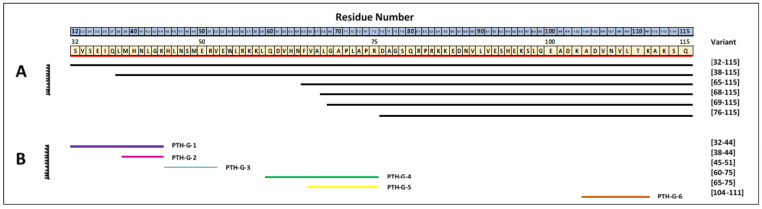
PTH variant map. (**A**) Truncated PTH variants identified previously. (**B**) Tryptic fragments were chosen for SRM- MSIA.

PTH is synthesized by the pituitary glands in a pre-pro form, which is cleaved to the active secreted hormone. The pre-PTH (115 amino acids) is trimmed by 25 amino-terminal residues (−6 to −31) to form pro-PTH (90 amino acids), which is further cleaved to the biologically active 84 amino acids and intact PTH (iPTH) (PTH1-84) [11]. PTH’s biological activity arises from PT binding, and several biologically active C-terminal and N-terminal fragments (proteoforms) have been identified in circulation. The term “proteoform” has recently gained recognition for protein derivatives arising from various processes, such as posttranslational processing, genetic polymorphisms, mutations, or truncations [12,13,14]. Several proteoforms of PTH have been identified in vivo, among which PTH (7–84) is the primary proteolytic PTH fragment produced by actions of cathepsins B and H. The current method of regulation for these proteoforms is as yet unidentified. Previously considered inert, these proteoforms are now known to have biochemical actions distinct from the active 84 amino acid hormone [15]. Recent studies indicate that the levels of PTH (7–84) and other amino-truncated PTH fragments increase considerably in diseases such as renal failure, chronic heart diseases, and bone disorders [16,17,18]. Moreover, a recombinant form of human PTH (1–34) was the first anabolic drug approved for the treatment of osteoporosis [19]. However, the precise identity of various PTH fragments and their clinical associations remains uncertain (Figure 1).

The quantitation of PTH is traditionally conducted using immunoassays to diagnose primary hyperparathyroidism and hypoparathyroidism and monitor disorders of the bone. PTH assays based on first-generation immunoassay utilize a single polyclonal antibody that targets PTH’s C-terminal or midterminal part. Since these assays measure several biologically inactive C-terminal fragments, specific PTH assays, such as second- and third-generation assays that differentiate between different truncated forms of PTH, are used instead of this approach [20,21]. Second-generation assays—termed intact PTH assays—suffer from interferences from multiple circulating fragments. Recently, third-generation assays were developed, which were considered far more specific than their previous counterparts and were considered to differentiate between these truncated forms [22,23]. Although serum PTH determination assays have significantly advanced, several limitations are associated with the immunometric generations of assessment PTH fragments. These limitations include difficulties in accurately quantifying specific pieces, the possibility of interference from non-PTH molecules, and the requirement for standardized assays to enhance reliability in clinical interpretations. The task of standardization was taken up by the International Federation of Clinical Chemistry (IFCC) working group that is working on PTH standardization and has provided the perspectives and priorities for the improvement of PTH measurement [24,25]. Therefore, new analytical approaches are needed to assess known PTH variations accurately and evaluate additional potential proteoforms simultaneously. Previous studies have utilized mass spectrometry to identify the different proteoforms of PTH in the plasma and serum [26,27,28,29,30].

Mass spectrometric immunoassay (MSIA) has recently gained significant attention in bioanalysis and biomarker discovery due to its exceptional specificity and sensitivity [31,32]. MSIA is a powerful technique that combines the strengths of immunoassay and mass spectrometry (MS) detection. The first demonstration of MSIA with matrix-assisted laser desorption/ionization (MALDI)-MS method was used to detect myotoxin α, utilizing antibodies that were derivatized onto agarose beads [33]. In addition, various proteoforms have been identified and quantified by validated MSIA approaches, such as apolipoprotein A-I, apolipoprotein C-III, insulin-like growth factor 1, and serum amyloid A [34,35,36,37].

In this study, we describe MSIA, specially tailored for detecting PTH proteoforms, and assess additional microheterogeneity in PTH related to vitamin D deficiency among patients with diabetes mellitus.

## 2. Materials and Methods

### 2.1. Materials and Chemicals

The mouse anti-parathyroid hormone (PTH) antibody, clone 3H9 (Cat. No MA1-83384), was obtained from Thermofisher Scientific (Waltham, MA, USA). The 20X modified Dulbecco’s Phosphate buffered saline (PBS) buffer (Cat. No. 28344) was obtained from Thermofisher Scientific, 20X modified Dulbecco’s Phosphate buffered saline Tween-20 (PBST) buffer (Cat. No. 28346) was obtained from Thermofisher Scientific, MSIA D.A.R.T Protein A/G 96 tips (Cat. No. 991PRT15) (Thermo Scientific, Waltham, MA, USA), and Finnipipette Novus Multichannel Electronic Pipette and stand (Thermo scientific). Acetonitrile (ACN), methanol (MeOH), formic acid (FA) dithiothreitol (DTT), iodoacetamide (IAA), and ammonium bicarbonate (ABC) were purchased from Sigma-Aldrich (St. Louis, MO, USA). RapiGest SF MS detergent was purchased from Waters Corporation (Milford, MA, USA). Both labeled (13C6, 15N2 labeled Lys and 13C6, 15N4 labeled Arg) and unlabeled peptides were synthesized by Biomatik Corporation (Kitchener, ON, Canada) with a minimum purity of 95%, aliquoted in 5 mg vials as dried pellets.

### 2.2. Subjects and Blood Samples

#### 2.2.1. Ethical Approval and Participant Recruitment

All study procedures were followed as per ethical standards of the Declaration of Helsinki and the Universal International Conference on Harmonization-Good Clinical Practice (ICH-GCP) guidelines laid down for human participants. Samples were collected from the participants after obtaining the necessary approval from the Institutional Review Board College of Medicine, King Saud University Hospital (No E-16-1752) and after obtaining written informed consent.

#### 2.2.2. Participant Recruitment and Sample Collection

We recruited forty patients with T2DM whose primary physician followed the King Saud University Medical City outpatient clinics. All patients had T2DM and no associated comorbidities. Samples were collected after a 10 h fast in EDTA tubes (Vacutainer, BD Biosciences, San Jose, CA, USA) by venipuncture. After centrifugation (15 min, 3000× *g*), plasma was separated, divided into several aliquots, and stored at −80 °C for further analysis. Routine laboratory investigations and estimations for 25 (OH) Vit D levels were carried out at the central biochemistry lab at KKUH. Patients with T2DM were assessed for their Vit D levels and grouped according to their vitamin D status. Patients with Vit D more than 75 nmol/L were considered as T2DM with normal vitamin D (DN, n = 20), and those with VitD levels less than 50 nmol/L (DD, n = 20) were considered as T2DM with deficient VitD.

### 2.3. Signature Peptide Selection

The protein sequence of the human parathyroid hormone (PTH) from the species homo sapiens, identified by the accession number P01270, was acquired from the protein database of the National Center for Biotechnology Information (NCBI). To generate potential signature peptides, trypsin mediated in silico digestion was performed using the PeptideMass tool available on the Expasy bioinformatics resource portal [38]. The peptides were subsequently analyzed using the Basic Local Alignment Search Tool (BLAST) to compare them with other protein sequences in the NCBI database and verify their distinctiveness.

Selecting signature peptides involved a multi-step process. First, each signature peptide length range was between 5 and 25 amino acids for the best performance in experiments. Second, water solubility assessments were performed using Innovagen’s peptide calculator (https://pepcalc.com/). Thirdly, the ProtParam tool (http://web.expasy.org/protparam/, accessed on 1 June 2023) was used to monitor peptide stability. Fourthly, the SIM alignment tool was utilized to confirm that all chosen peptides belonged exclusively to PTH and did not match with any other proteins present in the sample. Finally, posttranslational modifications (PTMs), including methylation, glycosylation, and phosphorylation, were verified using the UniProt and Phospho.ELM databases to ensure accuracy when measuring target proteins. Six distinct peptides, ^32^SVSEIQLMHNLGK^44^ (PTH-G1), ^38^LMHNLGK^44^ (PTH-G2), ^45^HLNSMER^51^ (PTH-G3), and ^60^DQVHNFVALGAPLAPR^75^ (PTH-G4), ^65^FVALGAPLAPR^75^ (PTH-G5), and ^104^ADVNVLTK^111^ (PTH-G6), were synthetically produced for the preparation of calibration curves. These peptides were produced in regular and stable-isotope-labeled forms, serving as internal standards (ISs). The materials, whose purity was greater than 95% as determined by high-performance liquid chromatography (HPLC), were subjected to peptide sequence identification. This analysis focused specifically on determining the location of the labeling using high-resolution mass spectrometry (HRMS). The supplier certifies the quality of the standard materials, providing HPLC chromatograms and MS spectra for each standard material.

### 2.4. MRM Transition Development

#### 2.4.1. Stock and Working Solution Preparation

For each labeled and unlabeled peptide, a 1.0 mg/mL stock solution in H_2_O was prepared. To tune the mass spectrometric conditions and optimize the chromatography, a 10.0 µg/mL working solution for each peptide was diluted in mobile phase A:B (0.1% FA in H_2_O:0.1% FA in 50% (*v*/*v*) ACN:MeOH).

#### 2.4.2. LC-MS/MS Analysis

As previously described in our prior study [39], a 10.0 ug/mL working solution of each labeled and unlabeled peptide was introduced into an LC-MS system (Waters Acquity UPLC coupled with XEVO TQ-MS (Waters Corporation, Milford, MA, USA)), using positive-ionization electrospray (+ESI) at a 0.3 mL/min flow rate. Chromatographic separation occurred via ultra-high-pressure liquid chromatography UHPLC BEH C18 columns (1.7 μm, 2.1 × 100 mm). The chromatographic separation was performed using a gradient elution profile. The flow rate was set to 0.3 mL/min throughout the run. The initial composition of the mobile phase was 90% solvent A (0.1% formic acid in H_2_O) and 10% solvent B (0.1% FA in 50% (*v*/*v*) ACN:MeOH). After 1 min, a linear gradient was applied over 10 min, shifting the composition to 10% solvent A and 90% solvent B. Afterward, the composition returned to the initial conditions (90% solvent A and 10% solvent B) within 1 min. The mobile phase remained at this composition for the final 3 min, making the total run time 15. The injection volume was set at 10 µL. Modifications were made to the general mass spectrometry (MS) parameters for LC-MS/MS. The ion source desolvation temperature was maintained at 450 °C. The flow rates for the ion source desolvation and cone gasses were set at min900 L/h and 50 L/h, respectively. The MS capillary and cone voltages were also set at 2 kV and 40 V, respectively.

### 2.5. Preparation of Plasma Sample and Enrichment Using MSIA DARTs

**Figure 2:**
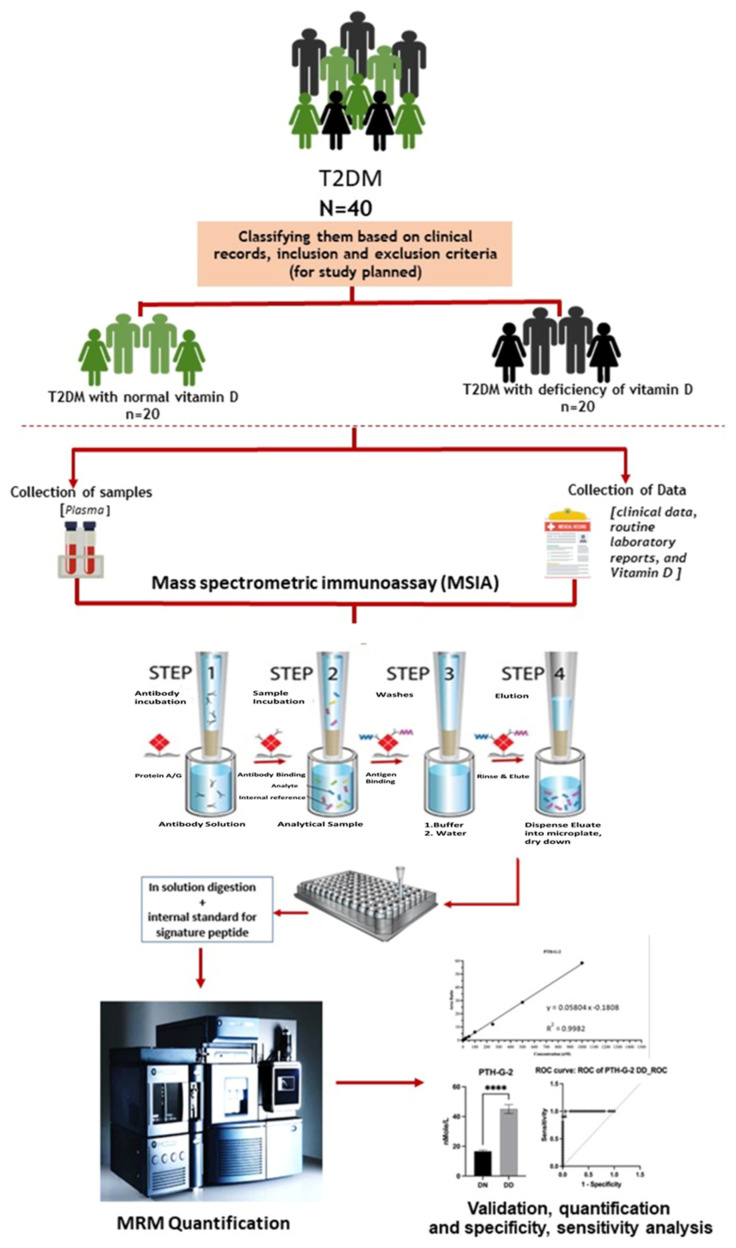
Experimental workflow for mass spectrometric immunoassay PTH. ****; *p* < 0.0001.

The patient’s samples were taken and prepared according to the Thermo Scientific protocol using protein A/G MSIA Disposable Automation Research Tips (DARTs) to obtain the proteins of interest. The Novus i multichannel pipette was loaded with protein A/G MSIA™-Tips, and repeated cycles of aspiration and dispersion were carried out, as outlined in Table 1, for the immunopurification of the target protein. Briefly, the plasma samples were diluted ten times with PBST, and then the 15 μL from this was mixed with 135 μL of PBS (100×) and added into the column. The tips were conditioned with PBS before binding 100 μL (0.05 mg/mL) of the mouse anti-PTH polyclonal antibody, clone 3H9 (100× plasma) that identifies the intact PTH peptide at the epitopes 1-34 and 1-84 (Cat-MA1-833384, Thermofisher, Waltham, MA, USA) to the protein’s A/G immobilized on the monolithic adsorbent surface (Figure 2). The target analytes (natural PTH proteoforms) were extracted from plasma by forming an antibody–antigen complex. Unbound antibody residues were removed with PBS and water after four consecutive washing steps. The natural PTH proteoforms from the samples were then eluted into tubes using an elution buffer (40% acetonitrile and 0.4% TFA in distilled water) (Table 1). They were dried using a speed vacuum centrifuge.

#### 2.5.1. In-Solution Tryptic Digestion

The tryptic digestion protocol was carried out by initially dissolving the proteins in 40 μL ammonium bicarbonate (50 mM) followed by incubation in the presence of dithiothreitol (DTT) (Sigma, St. Louis, MO, USA) (10 mM) at 56 °C for 1 h to reduce the disulfide bonds. Protein samples were next alkylated with iodoacetamide (IAA) (Sigma, St. Louis, MO, USA) (20 mM) at room temperature in the dark for another hour and were then digested using trypsin (Promega, Madison, WI, USA) in a 1:50 trypsin-to-protein ratio and incubated at 37 °C overnight. Lastly, 7 µL of 10% formic acid was added to stop the reaction.

#### 2.5.2. Solid-Phase Extraction (SPE)

Solid-phase extraction (SPE) was used to purify and concentrate the tryptic peptides following our standard protocol [40]. The Oasis HLB 1 cc 30 mg cartridge (Waters Oasis PRiME, Milford, MA, USA) was first conditioned with 100% methanol and then dH_2_O to wet the sorbent. Before running the samples through the sorbent, they were mixed with labeled peptides and diluted using a loading buffer (0.1% FA in dH_2_O). Unwanted substances were washed with dH_2_O twice. Tryptic peptides were eluted using a solution comprising 0.1% formic acid (FA) in 50% acetonitrile (ACN). Following drying using the Savant(tm) DNA Concentrator SpeedVac (Thermo Scientific, USA), dried extracts were reconstitution in a 100 μL mixture consisting of 90% A (0.1% formic acid in H_2_O) before being analyzed using LC-MSMS.

### 2.6. LC-MS/MS Method Validation Protocol

The validation study followed the FDA guideline [41,42], and the samples were analyzed on LC-MSMS, as detailed in Section 2.4.2.

#### 2.6.1. Linearity Verification

A mixture of unlabeled peptides with a concentration of 20 µg/mL was used to generate a calibration curve (0.1–1000 nM) and QC samples (1.5, 35, and 75 nM) prepared in an extracted matrix. This matrix was prepared from pooled serum samples, where the proteins were precipitated with an equal volume of acetone, and the pellets were resuspended with PBS buffer and digested tryptic. A constant amount (20 µL) of internal standard (20 µg/mL) was added to each sample and was run on the method detailed in Section 2.4.2. Calibration curves for peptides were generated over three consecutive days and executed to evaluate linearity. The peptide signals, expressed as area ratios (standard area/internal standard area, IS), were plotted on the y-axis. In contrast, the nominal concentrations of the standard solutions were plotted on the x-axis. The linearity of the peptide signals was determined through regression analysis, measuring the correlation coefficient (R^2^) and the precision and accuracy at different calibration levels.

#### 2.6.2. Limits of Detection

Three-day calibration curves provided an opportunity to establish limits of detection (LOD) and lower limits of quantification (LLOQ) for each peptide. LLOQ was defined as the lowest point on a calibration curve with signal at or exceeding 10× standard deviation of blank signal; S/N ratios were at least three or ten, depending on the concentration level for both concentrations; accuracy should range between 80 and 120% with daily variance less than 20%. Consequently, LLOD was adjusted so as to achieve an S/N ratio ≥3.

#### 2.6.3. Inter- and Intraday Validation

Three quality control (QC) concentration levels—low (LQC), medium (MQC), and high (HQC)—were utilized to evaluate the precision and accuracy of the method. This involved the analysis of six samples from each QC level, both on the same day, to assess intraday precision (repeatability) and over three different days for interday precision (reproducibility) to determine repeatability. The signal variability in this study was evaluated using the percent relative standard deviation (RSD). For each quality control (QC) sample, the intraday precision was calculated by the following formula: [(standard deviation of measured QC concentrations on a given day x)/(mean of measured concentrations on day x)] × 100%. Similarly, the interday precision was calculated as follows: [(standard deviation of measured QC concentrations over three days)/(mean of these measured concentrations over the three days)] × 100%. This study aimed to maintain a day-to-day variation below 20%. Intraday accuracy for each QC was calculated using the following formula: [(mean measured concentration)/(nominal concentration)] × 100%. The interday accuracy calculation followed the formula: [(mean measured concentration over three days)/(nominal QC concentration)] × 100%. For calibrators, accuracy within the 80–120% range for the limit of quantitation (LOQ) and 85–115% for other standard levels was considered acceptable.

#### 2.6.4. Stability of Peptide

Three quality control (QC) samples (high, medium, and low) were extracted and stored at different temperatures, such as room temperature (RT), refrigeration (4 °C), and freezing (−20 °C), to test the storage stability of selected signature peptides. These conditions were compared with those of freshly prepared QC samples. The assessment of storage stability for each QC level involved determining the molecular stability. This was accomplished by multiplying the average area ratio of the analyzed sample with that of the fresh sample and multiplying the result by 100%.

#### 2.6.5. Carryover

The highest standard was injected six times to assess the assay carryover, followed by injecting three blank samples. Carryover should not exceed 20% of LLOQ [43].

### 2.7. Data and Statistical Analysis

Statistical data analysis was performed using the Statistical Package for Social Sciences (SPSS) version 26 (SPSS Inc., Armonk, NY, USA) for the clinical variables between the two groups, and a *p*-value of less than 0.05 was considered statistically significant.

The LC-MS/MS MassLynx software, version 4.1 (Waters Corporation, Milford, MA, USA), was used to acquire, process, and visualize the data. The data for quantitative signals were displayed as mean values and SD. The protein levels of PTH G1-6 in patients with T2DM associated with vitamin D deficiency were compared to those in a group with normal vitamin D levels LC-MS/MS. Statistical analysis was performed using GraphPad Prism 6.01 (La Jolla, CA, USA), and *p*-values of less than 0.05 were considered significant.

## 3. Results and Discussion

### 3.1. Basic and Biochemical Characteristics

The basic characteristics and results of the biochemical parameters of the patients are described in Table 2. All the patients in the study had T2DM with either normal vitamin D levels or vitamin D deficiency. The mean age of patients with vitamin D deficiency was 45.3 ± 10.6, while those with normal vitamin D levels were 50.3 ± 13.2. There was no significant difference in the ages between the two groups and other biochemical parameters. A significant difference was noted only in the vitamin D levels between the groups (*p* < 0.01).

### 3.2. Signature Peptide Selection and LC-MS/MS Method Development

The protein sequences and isoforms of PTH (UniProt ID: P01270) were obtained from the human UniProt-FASTA database. Following the performance of in silico tryptic digestion on the PTH using PeptideMass, we could select signature peptides, which we confirmed with Skyline Software (version 19.1, Washington, DC, USA). The PTH’s signature peptides were chosen according to well-established criteria in the Methods section [39].

Table S1 details the characteristics of PTH-specific signature peptides selected for absolute quantification purposes, with light and heavy signature peptides acting as standards and internal standards (ISs). Calibration curves were constructed to measure signals using standard reference materials; furthermore, heavy isotopically labeled peptides have identical amino acid sequences and physical/chemical properties as the light peptides to maintain consistent ionization efficiency between unknown samples and calibration curves [44].

In this study, an internal standard (IS) version of each peptide was utilized, co-eluting at a similar retention time [45]. This approach corrected various analytical discrepancies, including differences in ionization efficiency, suppression, and the normalization of the signal. Thus, it helped determine concentrations using a calibration curve, where the response is based on the area ratio of the signature peptides to their internal standard [46].

### 3.3. Validation of LC-MS/MS Method

The method validation was performed in accordance with the guidelines set by the FDA [41,42].

#### 3.3.1. Curve Linearity

A calibration curve is used to set the quantification range and linearity of measurements for each of the chosen PTH signal peptides that correspond to each PTH variant (aa32–44, aa38–44, aa45–51, aa60–75, aa65–75, and aa104–111).

**Figure 3:**
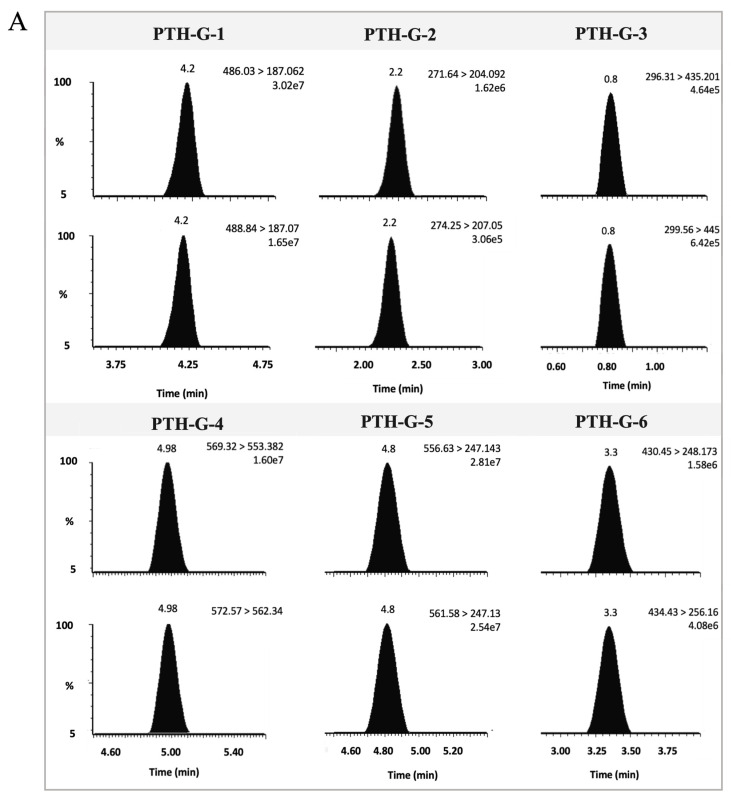

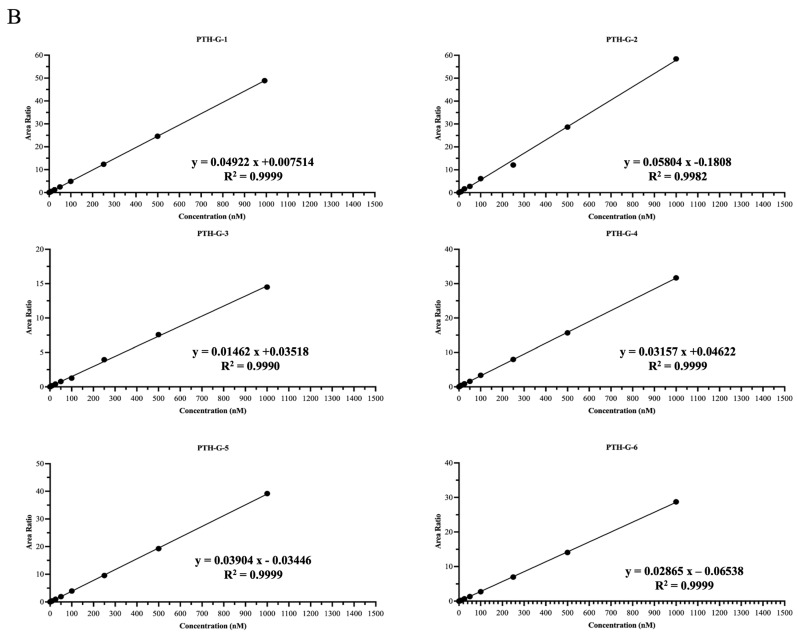
Representative extracted ion chromatography (C = 1000 nM) and calibration curves: (**A**) extracted ion chromatograms for the quantitative transitions for PTH-G-1 SP (486.03 > 187.06), PTH-G-2 SP (271.64 > 165.97), PTH-G-3 SP (296.31 > 435.2), PTH-G-4 SP (569.32 > 553.38), PTH-G-5 SP (556.63 > 247.14), PTH-G-6 SP (430.45 > 248.17), and their labeled internal standard transitions (488.84 > 187.07, 274.25 > 207.05, 299 > 445, 572 > 562.34, 561 > 247.13, 434 > 256.16, respectively); (**B**) calibration curves of PTH-G-1, PTH-G-2, PTH-G-3, PTH-G-4, PTH-G-5, and PTH-G-6.

Figure 3A,B show the extracted ion chromatograms of the PTH signature peptides (1 to 6), along with their labeled internal standards. The calibration curves for PTH variants exhibited excellent linearity over the range of 2.0 to 1000 nM with good linearity (r^2^ = 0.9999, 0.9982, 0.9990, 0.9999, 0.9999, and 0.9999, respectively).

#### 3.3.2. Specificity and Sensitivity

Table S2 summarizes the findings of the method’s sensitivity evaluation, which was conducted according to the procedures outlined in the Methods Section 2.

PTH signature peptides were assessed over three days with an LLOQ of 2.0 nM, which was confirmed through triplicate measurement over multiple trips in triplicate, along with method linearity analysis that yielded coefficients of determination (R^2^) values between 0.9999, 0.9982, 0.9990, 0.9999, 0.9999 and 0.9999. 

The absence of interferences with identical MRM transition and retention times characterizes specificity. The method’s specificity was assessed using LC-MS/MS. Furthermore, the blank sample from the carryover experiment described in the Methods section did not show any significant signal, amounting to less than 20% of the LLOQ.

#### 3.3.3. Inter- and Intraday Precision

Accuracy and precision were measured for PTH variants by analyzing six replicates of LQC, MQC, and HQC samples (25 nM, 150 nM, and 750 nM, respectively) on the same day (intraday) and three other days (interday) to meet the acceptance limits set by ICH; interday variability for selected signature peptides ranged between 15 and 17% while accuracy ranged between 80 and 120% among the quality controls (Table S3) [31]. Intraday accuracy ranged between 95.69 and 104.07%, with precision levels below 15 (Table S4).

#### 3.3.4. Peptide Stability Study

Three QC samples were used at three different concentrations (1.5, 35.0, and 75.0 nM) to evaluate the stability of the PTH signature peptides for the six variants. A stability assessment was conducted under various storage circumstances, including 24 h at room temperature, one week at 4 °C, two weeks at −20 °C, and one month at −20 °C. The stability of the selected signature peptides varied between 76.7% and 113.1% (Table S5).

### 3.4. Evaluation of Clinical Samples

**Figure 4:**
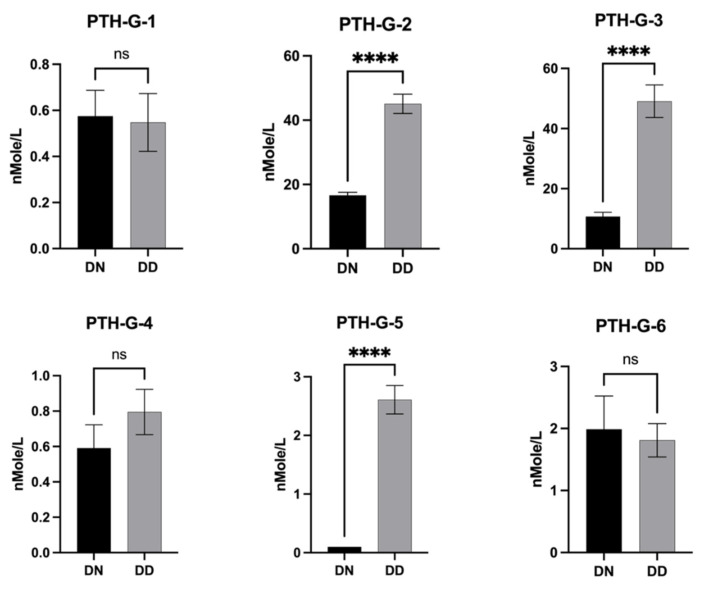
Bar graph of PTH-G-1, PTH-G-2, PTH-G-3, PTH-G-4, PTH-G-5, and PTH-G-6 levels in the serum of patient samples who were diabetic or had vitamin D deficiency (DD), and normal levels (DN). ****, *p* < 0.0001, ns; nonsignificant.

PTH variant concentrations were measured using their corresponding signature peptides (PTH-G-1-G-5). There was a significant increase in PTH-G-2 (aa38–44), PTH-G-3 (aa45–51), and PTH-G-5 (aa65–75) among the patients with T2DM associated with vitamin D deficiency compared to the normal vitamin D group (*p* < 0.0001) (Figure 4).

**Figure 5:**
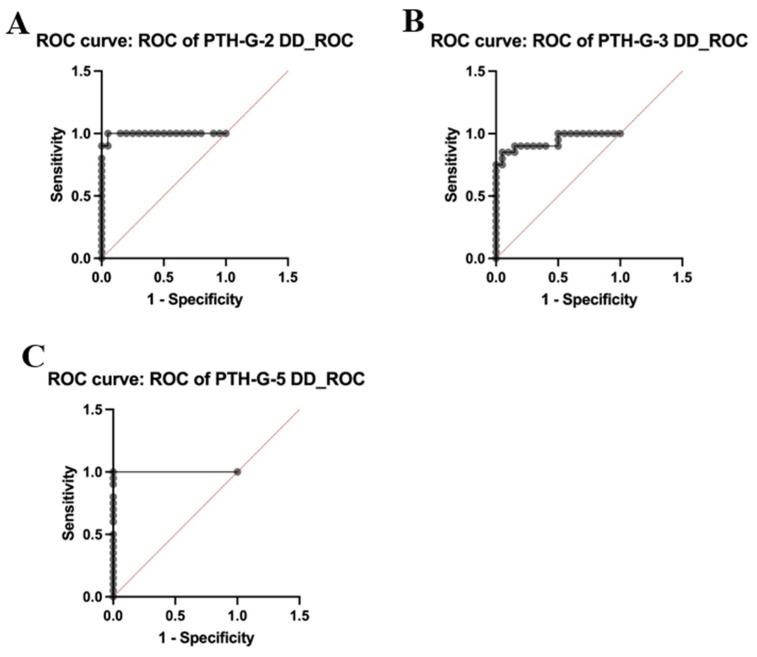
The receiver-operating characteristic (ROC) curve of (**A**) PTH-G-2, (95% CI, 0.9818–1.000), (**B**) PTH-G-3, (95% CI, 0.8629–1.000), and (**C**) PTH-G-5 (95% CI, 1.000–1.000) serum levels of patient samples who were diabetic or had vitamin D deficiency (DD).

The diagnostic values of PTH-G-2, PTH-G-3, and PTH-G-5 were assessed using receiver-operating characteristic (ROC) curves, as shown in Figure 5. The ROC curve plots the sensitivity (true positive rate) on the y-axis and the specificity (false positive rate) on the x-axis. The top left corner of the diagram represents the “ideal” position, characterized by zero false positives and one real positive. An ideal test has a maximum area under the receiver operating characteristic (ROC) curve (AUC) of one.

The area under the curve (AUC) values for PTH-G-2, PTH-G-3, and PTH-G-5 in individuals with T2DM and vitamin D deficiency, as well as those with T2DM and normal vitamin D levels, were 0.9950 (95% confidence interval [CI], *p* < 0.0001), 0.9375 (95% CI, *p* < 0.0001), and 1.000 (95% CI, *p* < 0.0001), respectively. Thus, PTH-G-2, PTH-G-3, and PTH-G-5 showed superior diagnostic and analytical capabilities in distinguishing between individuals with T2DM and vitamin D deficiency and those with T2DM and normal vitamin D levels.

The expression ratios for the peptides between people with diabetes with vitamin D deficiency (DD) and with normal (DN) levels ranged from 0.91 for ADVNVLTK (aa104–111) to 26.09 for FVALGAPLAPR (aa65–75) (Table S1).

Many clinical approaches use immobilized antibodies to quantify biomarkers, which are then detected by another antibody tagged with a fluorophore, radioisotope, or reporter enzyme. The capacity of antibodies to distinguish between different biomarker polymorphic variants and truncated proteoforms in a single analysis limits these approaches. This study reports the first use of MSIA to accurately identify and quantify PTH fragments between patients with T2DM and those with vitamin D deficiency and with normal vitamin D levels, and this method was successfully validated. Vitamin D deficiency is a risk factor for developing and worsening T2DM [47]. Lowering vitamin D levels, in turn, affects the levels of PTH and can lead to secondary hyperparathyroidism. This effect of alteration in PTH is not reflected in the conventional assays for PTH, which do not consider the presence of other PTH peptides that are biologically active. In this regard, our study shows the ability of different PTH fragments, including PTH-G-2 (aa38–44), PTH-G-3 (aa45–51), and PTH-G-5 (aa65–75), to discriminate between T2DM with vitamin D deficiency and T2DM patients with normal vitamin D levels. Moreover, vitamin D supplementation improves calcium absorption and reduces the stimulus for PTH secretion; this may alter the balance of PTH proteoforms. Specifically, with less demand for calcium regulation, there might be a reduction in the overall secretion of PTH, which could lead to a decrease in the levels of certain PTH fragments or changes in the relative proportions of these proteoforms. However, further studies are required to establish a definitive link between vitamin D supplementation and PTH proteoform distribution.

Despite MSIA’s power to distinguish between truncated proteoforms, this method has some limitations, such as the availability of high-quality antibodies, experimental design, and the absence of signals in mass spectra [48].

## 4. Conclusions

Utilizing this technique may provide significant benefits to clinical research laboratories, particularly in methods requiring the monitoring of numerous PTH variants or when analyzing samples that consist of a complex mixture of PTH-derived peptides and components resulting from the digestion of compounds within the sample matrix.

In addition, our findings suggest that PTH proteoforms have significant potential as biomarkers for monitoring calcium homeostasis, particularly in patients with T2DM who also experience vitamin D deficiency. Future research should focus on validating these proteoforms in larger cohorts to establish their clinical utility and potential for improving patient outcomes.

## Figures and Tables

**Table 1:** Pipetting scheme for PTH purification using a Thermo Finnpipette™ Novus i Multichannel Electronic Pipette equipped with Protein A/G MSIA™-Tips.

	1 Wash	2 Immobilization of Antibody	3 Wash	4 Antigen Capture	5 Wash	6 Wash	7 Wash	8 Wash	9 Elution
Liquid	PBS	100 μL (0.05 mg/mL) of the Mouse Anti Parathyroid Hormone (PTH) Antibody	10 mM PBS	150 μL of 100× Diluted Plasma in PBST	10 mM PBS	10 mM PBS	LC/MS Grade Water	LC/MS Grade Water	40% Acetonitrile and 0.4% TFA LC/MS Grade Water
Asp/Disp cycles	10×	250×	10×	100×	10×	10×	10×	10×	10×
Cycle volume (μL)	150	80	150	100	150	150	150	150	7
Total well volume (μL)	200	100	200	100	200	200	200	200	10

**Table 2:** Biochemical parameters of patients with T2DM and without vitamin D deficiency.

Parameter	Vitamin D Deficiency (n = 20)	Normal Vitamin D (n = 20)	*p*-Value
Male/Female (%)	60/40	70/30	NA
Age (years)	45.3 ± 10.6	50.3 ± 13.2	0.12
Glucose (mmol/L)	6.3 ± 0.8	7.2 ± 0.5	0.09
Urea (mmol/L)	4.3 ± 0.9	4.6 ± 0.8	0.26
Creatinine (umol/L)	71.1 ± 12.7	75.3 ± 12.0	0.074
Sodium (mmol/L)	132.6 ± 5.1	136.3 ± 5.9	0.13
Potassium (mmol/L)	4.2 ± 0.2	4.3 ± 0.4	0.40
Aspartate transaminase (IU/L)	44.8 ± 9.1	41.8 ± 4.5	0.38
Alanine transaminase (IU/L)	19.5 ± 5	18.8 ± 2.0	0.4
Alkaline phosphatase (IU/L)	97.6 ± 13.7	93.2 ± 28.9	0.3
Total cholesterol (mmol/L)	4.6 ± 1.0	4.9 ± 0.8	0.10
HDL cholesterol (mmol/L)	1.2 ± 0.2	1.4 ± 0.2	0.2
LDL cholesterol (mmol/L)	2.8 ± 0.9	3.1 ± 0.7	0.09
Triglycerides (mmol/L)	1.8 ± 0.6	2.5 ± 0.2	0.07
vitamin D (nmol/L)	27.7 ± 9.3	97.5 ± 22.9	<0.01 *

Values are presented as mean ± SD. * *p*  < 0.05.

## Data Availability

The datasets used and/or analyzed during the current study are available from the corresponding author on reasonable request.

## Supplementary Materials

The following are available online at https://www.mdpi.com/article/10.3390/proteomes12040030/s1. Table S1: List of PTH signature peptides and their internal standard details; Table S2: Linearity and sensitivity summary over three days.; Table S3: Interday validation summary; Table S4: Intra-day validation summary. SD: Standard deviation. Table S5: Summary of PTH peptides’ stability at different storage conditions for a determined period.

## Abbreviations

PTH	Parathyroid hormone
CLIA	Clinical Laboratory Improvement Amendments
MSIA	Mass spectrometric immunoassay
T2DM	Type 2 diabetes mellitus
VDRs	Vitamin D receptors
MeOH	Methanol
FA	Formic acid
ABC	Ammonium bicarbonate
ICH-GCP	International Conference on Harmonization-Good Clinical Practice
DN	Diabetic patients with normal vitamin D
DD	Diabetic patients with deficient vitamin D
IS	Internal standard
D.A.R.T.	Disposable Automation Research Tips
RSD	Relative standard deviation
QC	Quality control
