# Supplementary material for: Multiple Reaction Monitoring–Mass Spectrometric Immunoassay Analysis of Parathyroid Hormone Fragments with Vitamin D Deficiency in Patients with Diabetes Mellitus
Source: Proteomes. 2024 Oct 14;12(4):30. doi: 10.3390/proteomes12040030 (PMC11503337; doi:10.3390/proteomes12040030)
Supplement: Supplementary file 1 [file proteomes-12-00030-s001.zip › proteomes-3117707-supplementary.pdf]

Table S1: List of PTH signature peptides and their internal standard details, including the instrumental conditions.

Signature Peptide ID	Peptide sequence	Molecular weight (g/mol)	Precursor ion Q1 (m/z)[z]	Product ion Q3 (m/z)	Product ion type (z)	Type	Cone voltage (v)	Collision Energy (v)	Retention time (min)	Quantification Property
PTH-G-1	^{32}SVSEIQLMHNLGK^{44}	1455.5	486.03 [3]	159.06	Y3 (+2)	Standard	24	20	4.27	Qual
				187.06	B2 (+1)		24	15		Quant
	^{32}SVSEIQLMHNLGK^{44}	1463.7	488.84 [3]	159.07	Y3 (+2)	Internal Standard	24	20		Qual
				187.07	B2 (+1)		24	20		Quant
PTH-G-2	^{38}LMHNLGK^{44}	811.8	271.64 [3]	165.97	B4 (+3)	Standard	24	15	2.29	Quant
				204.09	Y2 (+1)		24	15		Qual
	^{38}LMHNLGK^{44}	820.0	274.25 [3]	165.97	B4 (+3)	Internal Standard	24	15		Qual
				207.05	Y2 (+1)		24	15		Quant
PTH-G-3	^{45}HLNSMER^{51}	886.1	296.31 [3]	304.19	Y1 (+1)	Standard	22	15	0.81	Qual
				435.2	Y3 (+1)		22	10		Quant
	^{45}HLNSMER^{51}	896.0	299.56 [3]	314.07	Y1 (+1)	Internal Standard	22	15		Qual
				445	Y3 (+1)		22	10		Quant
PTH-G-4	^{60}DQVHNFVALGAPLAPR^{75}	1705.1	569.32 [3]	277.24	Y5 (+2)	Standard	26	15	5.02	Qual
				553.38	Y5 (+1)		26	15		Quant
	^{60}DQVHNFVALGAPLAPR^{75}	1714.0	572.57 [3]	282.15	Y5 (+2)	Internal Standard	26	15		Qual
				562.34	Y5 (+1)		26	15		Quant
PTH-G-5	^{65}FVALGAPLAPR^{75}	1111.4	556.63 [2]	219.12	B7 (+3)	Standard	28	35	4.68	Qual
				247.14	B2 (+1)		28	20		Quant
	^{65}FVALGAPLAPR^{75}	1121.3	561.58 [2]	219.1	B7 (+3)	Internal Standard	28	20		Qual
				247.13	B2(+1)		28	20		Quant
PTH-G-6	^{104}ADVNVLTK^{111}	858.9	430.45 [2]	187.05	B2 (+1)	Standard	28	15	3.36	Qual
				248.17	Y2 (+1)		28	20		Quant
	^{104}ADVNVLTK^{111}	866.9	434.43 [2]	187.02	B2 (+1)	Internal Standard	28	20		Qual
				256.16	Y2 (+1)		28	20		Quant

Table S2: Linearity and sensitivity summary over three days.

Signature Peptide ID	LLOQ (n=6)			Slope (n=3)	Intercept (n=3)	R2 (n=3)
	nM	Inter-day Accuracy (%)	Inter-day precision (%)			
PTH-G1	0.10	93.1	6.0	0.0486	0.2720	0.9994
PTH-G2	0.10	101.2	7.3	0.0580	-0.1808	0.9982
PTH-G3	0.10	102.3	8.2	0.0136	0.0910	0.9974
PTH-G4	0.10	89.0	12.3	0.0347	0.0667	0.9998
PTH-G5	0.10	95.3	12.3	0.0376	-0.0262	1.0000
PTH-G6	0.10	96.8	3.6	0.0287	-0.0654	0.9999

Table S3: Inter-day validation summary.

Signature Peptide ID	QCL 1.5 nM (n=6)				QCM 35.0 nM (n=6)				QCH 75.0 nM (n=6)			
	Mean	SD	Accuracy %	Precision %	Mean	SD	Accuracy %	Precision %	Mean	SD	Accuracy %	Precision %
PTH-G1	1.51	0.06	100.94	4.12	33.71	0.52	96.32	1.55	79.54	1.02	106.06	1.28
PTH-G2	1.50	0.04	99.67	2.53	36.70	1.54	104.86	4.21	75.15	1.30	100.20	1.73
PTH-G3	1.45	0.16	96.58	11.21	32.85	2.06	93.86	6.26	71.63	2.76	95.51	3.86
PTH-G4	1.50	0.09	99.78	6.32	33.97	2.80	97.05	8.24	78.19	4.52	104.26	5.78
PTH-G5	1.56	0.16	104.07	10.31	37.42	1.25	106.90	3.35	79.04	1.13	105.38	1.43
PTH-G6	1.46	0.05	97.11	3.09	35.70	2.60	102.00	7.28	68.77	5.49	91.69	7.99

Table S4: Intra-day validation summary. SD: Standard Deviation

Signature Peptide ID	QCL 1.50 nM (n=18)				QCH 35.00 nM (n=18)				QCH 75.00 nM (n=18)			
	Mean	SD	Accuracy %	Precision %	Mean	SD	Accuracy %	Precision %	Mean	SD	Accuracy %	Precision %
PTH-G1	1.45	0.13	96.73	9.06	33.25	0.61	95.00	1.83	74.40	4.22	99.20	5.67
PTH-G2	1.51	0.11	100.93	7.28	35.16	2.59	100.46	7.35	72.85	7.31	97.13	10.04
PTH-G3	1.48	0.13	98.39	8.78	35.66	3.42	101.90	9.59	75.99	4.62	101.32	6.08
PTH-G4	1.44	0.11	95.69	7.84	33.91	3.15	96.89	9.28	75.12	6.94	100.16	9.24
PTH-G5	1.55	0.11	103.17	7.43	39.67	2.14	113.33	5.40	82.57	3.50	110.09	4.23
PTH-G6	1.46	0.16	97.33	11.19	34.04	3.12	97.27	9.15	70.07	5.31	93.43	7.58

Table S5: Summary of PTH peptides stability at different storage conditions for a determined period. QC: Quality Control, RT: Room Temperature.

Signature Peptide ID	QC (nM)	Stability (%)							
		Fresh (-80 °C)		RT (24 h)		4°C (1 Week)		–20 °C (1 Month)	
		Mean %	SD	Mean %	SD	Mean %	SD	Mean %	SD
PTH-G1	1.5	100	13.6	97.8	6.5	91.1	7.2	101.2	7.4
	35.0	100	4.7	102.7	1.7	81.3	13.5	100.5	3.3
	75.0	100	6.8	100.5	3.3	105.7	4.1	104.9	2.2
PTH-G2	1.5	100	2.7	89	6.2	92.4	8.3	96.9	15.2
	35.0	100	13.2	107.9	9.8	115.6	2.8	90.2	15.1
	75.0	100	9.6	91.6	7.6	87.5	13.8	90.2	1.1
PTH-G3	1.5	100	7.4	99.3	1.6	101.7	9.2	114.6	5.5
	35.0	100	9.4	111.2	12.2	94.5	18.9	113.1	15.9
	75.0	100	5.4	78.5	5.6	104.3	13.7	98.9	2.4
PTH-G4	1.5	100	10.9	99.2	0.6	105.9	6.9	105.2	4.4
	35.0	100	5.1	99.2	5.1	76.5	17.8	99.1	2.8
	75.0	100	3.1	76.7	2.2	86.3	3.5	91.8	13.5
PTH-G5	1.5	100	6.3	78.5	4.1	97.8	6.4	81.3	4.1
	35.0	100	9.8	99.1	8.3	102.7	9.8	105.7	8.3
	75.0	100	7.6	99.2	2.8	100.5	7.6	92.4	2.8
PTH-G6	1.5	100	1.6	76.7	13.8	89.8	1.6	105.6	13.8
	35.0	100	12.2	87.3	9.2	107.9	12.2	87.7	9.2
	75.0	100	5.1	101.7	13.8	94.5	5.6	81.3	18.9
